# Challenges in Tick-Borne Pathogen Detection: The Case for *Babesia* spp. Identification in the Tick Vector

**DOI:** 10.3390/pathogens10020092

**Published:** 2021-01-20

**Authors:** Grecia Martínez-García, R. Montserrat Santamaría-Espinosa, José J. Lira-Amaya, Julio V. Figueroa

**Affiliations:** Babesia Unit-CENID-Salud Animal e Inocuidad, INIFAP, Carr. Fed. Cuernavaca-Cuautla No. 8534, Col. Progreso, Jiutepec, Morelos C.P. 62550, Mexico; martinez.grecia@inifap.gob.mx (G.M.-G.); santamaria.rebeca@inifap.gob.mx (R.M.S.-E.); lira.juan@inifap.gob.mx (J.J.L.-A.)

**Keywords:** *Babesia* spp., ticks, detection, babesiosis

## Abstract

The causative agents of Babesiosis are intraerythrocytic protozoa of the genus *Babesia*. *Babesia* parasites are present around the world, affecting several mammals including humans, pets and livestock, hence its medical and veterinary relevance. *Babesia* spp. detection in its invertebrate host is a main point in understanding the biology of the parasite to acquire more knowledge on the host–*Babesia*–vector interactions, as increasing knowledge of the *Babesia* lifecycle and babesiosis epidemiology can help prevent babesiosis outbreaks in susceptible mammals. The aim of the present review is to highlight the newest findings in this field, based on a bibliographic compilation of research studies recently carried out for the detection of the main *Babesia* species found in tick vectors affecting mammalian hosts, including the different tick stages such as adult ticks, larvae, nymphs and eggs, as well as the detection method implemented: microscopic tools for parasite identification and molecular tools for parasite DNA detection by conventional PCR, nested-PCR, PCR-RFLP, PCR-RLB hybridization, real time-PCR, LAMP and RAP assays. Although molecular identification of *Babesia* parasites has been achieved in several tick species and tissue samples, it is still necessary to carry out transmission experiments through biological models to confirm the vectorial capacity of various tick species.

## 1. Introduction

Ticks and tick-borne diseases are one of the main medical, veterinary and economic problems worldwide. Ticks are ectoparasites with blood-sucking habits and high vector capacity, and parasitize amphibians, birds, mammals and reptiles. In addition to the damage caused per se due to blood intake, injection of toxins and skin damage due to the bite, ticks can transmit several pathogens, such as viruses, bacteria, protozoa and rickettsiae [1,2]. Currently, more than 900 species of ticks have been described worldwide, divided into four families: Argasidae, Ixodidae, Nuttalliellidae and Deinocrotonidae. The Ixodidae family represents around 700 tick species, several members of these family belonging to the genera *Dermacentor*, *Haemaphysalis, Hyalomma, Ixodes* and *Rhipicephalus*, and can transmit the pathogens causative of babesiosis disease [3,4,5].

Babesiosis is considered one of the main vector-borne diseases caused by intraerythrocytic parasites, just behind malaria disease whose etiological agent is transmitted by mosquitoes [6]. The vast distribution of *Babesia* parasites that affect domestic animals, in addition to the great economic losses due to the presence of babesiosis in tropical and subtropical regions of the world and joined to the zoonotic capacity of some *Babesia* spp., have made babesiosis a disease of public health and veterinary importance [7,8,9]. Babesiosis disease is caused by an intraerythrocytic protozoan parasite of the genus *Babesia*, belonging to the order Piroplasmidae and the phylum Apicomplexa. *Babesia* parasites infect red blood cells of humans, domestic and wildlife animals, and recently, have been reported in birds [6,8,10]. The first report about *Babesia* spp. was made by Victor Babes in 1888, when he discovered the parasite in the red blood cells of cattle and associated it as the cause of hemoglobinuria or red water fever in Romanian cattle [11], he also found this microorganism in erythrocytes of sheep affected by a disease called Carceag [12]. Later, Smith and Kilborne in the USA demonstrated for the first time that *Boophilus annulatus* ticks (now reclassified as *Rhipicephalus annulatus*) were the vectors responsible for transmitting “Texas Fever” disease (bovine babesiosis) [13]. Skrabalo and Deanovic [14] reported the first case of human babesiosis in a splenectomized young man in Yugoslavia; while in 1970, in the United States, a middle-aged woman with intact spleen and previously reported as healthy, presented *B. microti*-like parasites in the erythrocytes [15]. Since then, several cases in humans have been reported in Asia, Africa, Australia, Europe, and mainly, in the USA [16]. At present, more than 100 different *Babesia* species have been recognized around the world, parasitizing various mammals and birds, nevertheless this number is increasing due to new research in other possible vertebrate hosts. Previously, *Babesia* parasites had been taxonomically classified according to their phenotypic and lifecycle characteristics, but recently, molecular phylogeny studies using the 18S rRNA gene have helped to reclassify *Babesia* species in a more comprehensive way, including the *Babesia* parasites in two large groups: *Babesia sensu stricto* and *Babesia sensu lato* [6,17,18].

**Figure 1:**
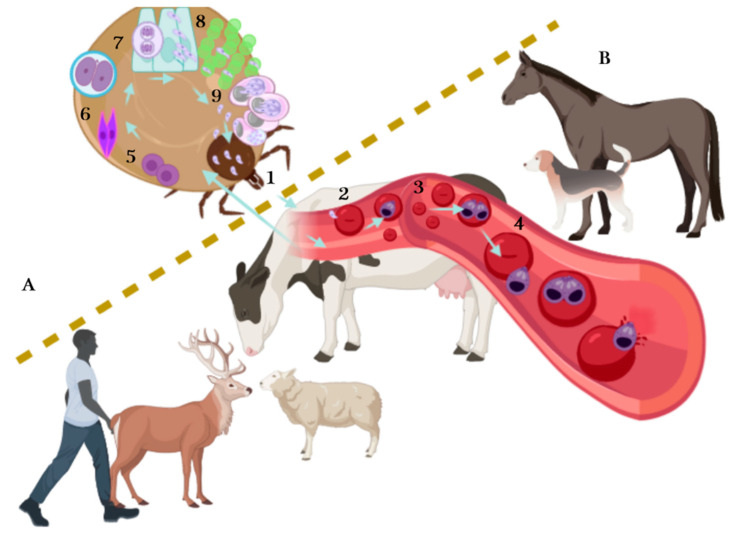
The lifecycle of *Babesia* spp. The lifecycle takes place in ticks (**A**) and vertebrates (**B**). Asexual reproduction is carried out in vertebrates: it begins when the infected tick feeds on the host and inoculates the infective phase of *Babesia*, the sporozoites (1). Sporozoites travel through bloodstream and invade red blood cells (RBCs) (2); once inside the RBCs, sporozoites become a trophozoite (3). Later, the merogony phase occurs, resulting in two or more merozoites (4), merozoites lyse the infected RCBs and continue invading new RBCs—some merozoites mature and turn into pre-gametocytes (beginning the gamogony phase). When ticks suck blood, healthy and parasitized RBCs are ingested, the pre-gametocytes present in RBCs develop into extracellular gametocytes (5), there is a fusion of male and female gametocytes and the ookinete is formed (6). The ookinete, also known as motile zygote, invades the intestinal cells of the tick helped by its arrowhead (7), and a meiotic division occurs giving rise to the kinetes. Kinetes travel through hemolymph and invade other tick tissues including ovaries and embryos in adult female ticks (8) and disseminate to salivary glands, where they develop into sporoblasts. The sporoblast remains inactive in salivary glands until it transforms into sporozoites (sporogony phase) (9), repeating the cycle when sporozoites are newly released to the mammalian bloodstream.

The lifecycle of *Babesia* spp. (Figure 1) takes place in ticks and vertebrates, where it reproduces sexually and asexually respectively, and includes a merogony, gamogony and sporogony phase [19]. The cycle begins when the infected tick feeds on the vertebrate host and at the same time, inoculates saliva infected with sporozoites, the infective phase of *Babesia.* Sporozoites travel through the bloodstream and penetrate the red blood cells. Later, the hemoparasite becomes a trophozoite, taking a ring shape: trophozoites divide by binary fission (merogony phase) in two or more merozoites (it depends on *Babesia* species), while merozoites lyse the erythrocyte and again invade a new erythrocyte. Gamogony begins in the vertebrate host where mature merozoites turn into pre-gametocytes. When ticks suck blood, healthy and parasitized (with the different stages of *Babesia*) erythrocytes are ingested equally, but only pre-gametocytes survive and develop into gametocytes (beginning the sexual phase) [20,21,22]. In the tick gut lumen, gametocytes become gametes, also known as Strahlenkörper or spiky-rayed bodies. The gametes fuse to form an ookinete (motile zygote), the ookinete arrowhead helps them to penetrate tick gut cells, where a meiotic division occurs giving rise to a new cell, the kinete. Kinetes travel through hemolymph and invade tick tissues, including the ovaries in adult female ticks and tick embryos (transovarial transmission); at the same time, kinetes disseminate to the salivary gland, where they develop into sporoblasts [17,20,22]. These new cells remain inactive in the cytoplasm of the salivary gland cells until the next tick generation or the next tick stage (after molting) attaches on a mammalian host, allowing transstadial transmission. Sporoblasts present in salivary glands produce 5000–10,000 infective sporozoites by a single alveolus during the sporogony phase and are finally released to the mammalian bloodstream [19], repeating the cycle. It is important to note that transovarial transmission does not occur in *Babesia sensu lato* species, while transstadial transmission occurs in *Babesia sensu lato* and *Babesia sensu stricto*. In addition, not all the tick stages can transmit the parasites, each *Babesia* species needs a specific time for sporoblast maturation [21]. On the other hand, the tick life stage while feeding on the mammal is not an impediment for it to acquire the parasite and, in some cases, the parasite multiplication can occur [22].

Different tests have been designed for the detection of *Babesia* spp. in its invertebrate and vertebrate host, based on microscopy, molecular and serological techniques, either for direct or indirect detection of the parasite. *Babesia* parasites can be detected by microscopy visualization in samples such as tick tissues (hemolymph, salivary gland, midgut, etc.) and host blood smears, although its use for clinical diagnosis is only suitable in symptomatic cases during the acute phases of the disease. The main drawback of this technique is the need for a qualified operator to find *Babesia* parasites due low levels of parasites present in the sample and, besides, who can discern between species. Molecular tools have shown high values of specificity and sensitivity, mainly PCR assays in its different formats such as conventional, hemi-nested and nested PCR. These assays are commonly followed by sequencing of PCR products obtained by sequence analysis, and most recently, by phylogenetic analysis. Other variants frequently used for this assay are PCR-RFLP, qPCR and RT-PCR. The LAMP and RAP assays are detection methods also based on the molecular identification and have been implemented to *Babesia* detection, but mostly in its invertebrate host. Whereas, the serological methods implemented in vertebrate hosts include: Indirect Fluorescent Antibody Test (IFAT), Enzyme-Linked Immunosorbent Assay (ELISA), Complement Fixation Test (CFT) and Immunochromatography Test (ICT) [23,24,25,26,27,28,29,30].

The identification of *Babesia* in its vector tick is necessary to acquire more knowledge on the host–*Babesia*–vector interactions. Additionally, the detection of *Babesia* parasites is required to determine the prevalence of these protozoa in the different tick species and tick stages, helping to know the transmission capacity of *Babesia* parasites, either during the transstadial or the transovarial transmission development. Likewise, planning and development of epidemiological surveys that allow to identify the probability of babesiosis outbreaks will in turn serve to perform successful strategies to prevent and control the pathogens and the diseases they cause. Recently, some *Babesia* species have been identified in ticks other than those commonly known as being its main vector; furthermore, recent climate change is a new factor to be considered for monitoring ticks, due to the presence of ticks in regions previously reported as tick-free, increasing the possibility for tick-borne disease outbreaks, including babesiosis.

The aim of the present review is to highlight part of the bibliographic compilation of research studies recently carried out for the detection of the main *Babesia* species in tick vectors affecting mammalian hosts, including the different tick stages, as well as the detection method implemented. The authors are aware that this review is not fully exhaustive and express their most sincere apologies to those researchers whose articles were not included.

## 2. Detection Methods for *Babesia* Identification

Several detection methods are used for *Babesia* spp. identification in ticks, these include mainly molecular and microscopical tools. For the detection of *Babesia* parasites DNA in the various tick species and different tick stages, several authors have decided to pool various amounts of eggs, larvae or nymphs, and even adult ticks (male or female), and then take the pools as a single sample. In general, pooled ticks either belong to the progeny of the same adult female or are collected from the same animal during sampling. Pooling is recommended to obtain a greater quantity of DNA that would allow the identification of *Babesia* DNA. Whereas, for the microscopic detection with the hemolymph test, only the hemolymph present in one of the tick legs is utilized to search for kinetes, although the presence of kinetes can also be determined in tick egg masses. The *Babesia* parasites considered in this review will be approached based on the host mammal they infect and according to the detection methods used. In Table 1, the different *Babesia* species are tabulated according to their principal tick-vectors, regarding their geographical distribution and the detection methods utilized for its identification in tick samples. Additionally, the primers sequences used in the cited studies are summarized in Table 2, along with the identification of the target genes and the expected amplicon lengths.

### 2.1. Bovine Babesiosis

The main *Babesia* species that have been reported as the causative agents of bovine babesiosis are *Babesia bovis*, *B. bigemina* and *B. divergens* (Table 1), these species elicit several important clinical signs and, in some cases, can cause death in cattle because of an inadequate diagnosis and timely treatment. Also, *B. divergens* is a hemoparasite of zoonotic importance. In addition to parasitizing cattle, *Babesia bovis* and *B. bigemina* can also be found in water buffalo, serving these as a reservoir, but they do not develop the clinical disease [7]. Other *Babesia* species with lower pathogenicity have been identified in cattle, such as *B. ovata* found in Asian cattle populations, which can cause severe anemia in immunosuppressed animals, hence the importance of its detection, as well as *B. major*, *B. orientalis* and *B. occultans* that can infect cattle [31] (Table 1). Bovine babesiosis is widely distributed in the world with a global prevalence of 29%, with the highest prevalence present in South America, where *Babesia bigemina* is the most commonly found parasite [32,33]. Overview of *Babesia* species detected in Ixodidae ticks referenced in this study and the detection methods used is shown in Table 1 [34,35,36,37,38,39,40,41,42,43,44,45,46,47,48,49,50,51,52,53,54,55,56,57,58,59,60,61,62,63,64,65,66,67,68,69,70,71,72,73,74,75,76,77,78,79,80,81,82,83,84,85,86,87,88,89,90,91,92,93,94,95,96,97,98,99,100,101,102,103,104,105,106,107,108,109,110,111,112,113,114,115,116,117,118,119,120,121,122,123,124,125,126,127,128,129,130,131,132,133,134].

#### 2.1.1. Microscopy Tools

The detection of *Babesia* parasites can be accomplished by microscopical examination of hemolymph and eggs squashes obtained from engorged adult female ticks collected from infected animals [23,33]. In a study conducted in calves and cows in an endemic zone of *Rh. microplus* in Brazil, it was possible to correlate the quantity of kinetes present in hemolymph smears from adult engorged female ticks with the progeny larval hatching rate. After the female ticks laid their eggs on the 15th day, a leg of each adult female tick was sectioned and a hemolymph smear was made. The egg smears were made by crushing 100 eggs derived from a single female tick, while another 100 eggs were incubated to determinate the hatching rate. Results showed that for each kinete found in the hemolymph smear, the hatching rate decrease by 0.57%. Also, authors found that the *Babesia* frequency in hemolymph and eggs was higher in ticks collected from calves than in ticks collected from cows [34]. The efficiency of tick acquisition and transovarial transmission of *B. bovis* was assessed by light microscopy examination of hemolymph samples [35,36]. It was found that bovine blood with high parasite levels in acutely infected animals is directly related to high kinete levels found in replete females following acquisition feeding. There was a positive correlation between the highest parasite levels in the blood and the percentage of engorged females containing high levels of kinetes in hemolymph samples [35]. However, female ticks that fed to repletion on persistently infected calves did not show detectable kinetes by light microscopy. Even though there were no parasites detected by light microscopy in tail capillary smears from persistently infected calves during female tick acquisition, female ticks can acquire the parasite and pass it trans-ovarially to larval offspring [36].

**Figure 2:**
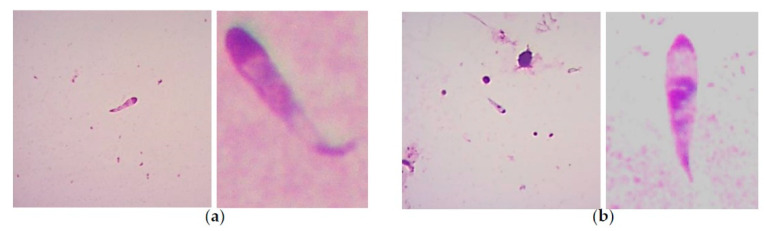
Giemsa-stained smears showing examples of bovine *Babesia* spp. kinetes found in tick hemolymph samples of *Rh. microplus* engorged females: (**a**) *B. bovis* kinetes can present a curved or semi-curved tail, but this is not always the case, with an anterior position nucleus (that generally could be found at the middle of the cells) that can have a mean length of 14.30 ± 0.922 μm and a mean width of 3.33 ± 0.315 μm. (**b**) *B. bigemina* kinetes show a straight tail and a median nucleus position, and present a smaller size to *B. bovis* kinetes (mean length of 11.27 ± 0.900 and width of 2.24 ± 0.287 μm). Kinetes size of both species can have variations depending on the strains examined and the geographic origin, added to the fact that *B. bovis* curved tail it is not always present, that makes the standardizations of the criteria difficult to be followed for microscopic identification.

In another study conducted on *B. bigemina* infecting various genetic groups of cattle, *Babesia* spp. kinetes were found in the hemolymph obtained from *Rh. microplus* female ticks that engorged on calves. Regardless of the genetic group of the tick-infested cattle, a range of 0.13–3.2 kinetes per microscopic field was observed [37]. It is important to point out that in the studies conducted by Oliveira et al., the identification of *Babesia* species (either *B. bigemina* or *B. bovis*) was not achieved, due to the difficulty represented by the microscopic identification of *Babesia* kinetes. It has been established that the usefulness of assessing the gross morphological features to differentiate *Babesia bovis* and *Babesia bigemina* kinetes in a hemolymph sample from engorged female ticks is dubious [33]. In addition, it was demonstrated that tick hemolymph infection is sometimes undetectable by light microscopy examination, but transmission to larval progeny occurs as demonstrated in a tick larvae-infested bovine [36]. The difficulty to define *Babesia* species based on kinete morphology determined in hemolymph samples from engorged female *R. microplus* ticks infected with *B. bovis* or *B. bigemina* can be better exemplified in Figure 2. Therefore, nowadays, it is imperative to apply the overly sensitive molecular tools currently available in these types of studies to determine, besides the species, the vector competence for single or dual *Babesia* infection in ticks.

#### 2.1.2. Molecular Tools

The molecular identification of *B. bovis* and *B. bigemina* through nested-PCR is one of the main utilized techniques, due to the high sensibility and specificity achieved. In several studies, the primers designed for the amplification of the *SpeI*-*AvaI* restriction fragment for *B. bigemina* (BiIA/BiIB and BiIAN/BiIBN) and for the amplification of a *B. bovis* gene encoding a 60 kDa merozoite surface protein (BoF/BoR and BoFN/BoRN) have been successfully used in the past for detection of both parasite species in tick samples (Table 2). These nPCR assays were originally designed to identify *Babesia* parasites in red blood cells, however, several studies have found their utility for *B. bovis* and *B. bigemina* detection on samples derived from adult, eggs and larvae tick stages [38,39]. The first study carried out to detect *B. bigemina* and *B. bovis* in adult ticks by PCR using the primers described above was published in the year 2000 [40]. The authors found relatively high infection rates in collected ticks: the 5% *B. bigemina* infection rate in *B. decoloratus* allowed to oviposit before PCR analysis, whereas 60% were positive with primers for *B. bovis*. Similarly, in *B. microplus* allowed to oviposit, 69% were positive for *B. bovis* DNA and 12% were positive for *B. bigemina* [40]. Next, a molecular epidemiological study evaluating the nPCR assays mentioned previously was conducted in Brazil, where the authors identified higher *Babesia* positivity in *Rhipicephalus microplus* ticks collected from calves than in ticks removed from cows, as well as a higher percentage of *B. bigemina*-infected ticks as compared to the *B. bovis*-infected ticks. In addition, the eggs oviposited by the collected ticks were also evaluated, and similarly, *B. bigemina* showed a higher presence in eggs derived from the ticks present in calves, whereas the infection rate was low for both parasites in ticks derived from cows. Of note is that DNA extraction was made from 20 mg of eggs out of every single tick collected. Despite the high sensitivity shown by nPCR, the authors reported a false-negative host vertebrate sample, since they found ticks positive for *B. bovis* or *B. bigemina* but derived from nPCR-negative animals to the corresponding species [41]. In another study, PCR based on primers BiIA/BiIB and BiIAN/BiIBN has been used in India to determine the *B. bigemina* molecular prevalence in egg masses and unfed larvae from *Rh. microplus* ticks (Table 2). For both types of samples, a pull of 100 eggs/larvae derived from the same progeny were used for DNA extraction. In that study, nPCR was compared to conventional PCR and microscopy detection from hemolymph and squashed egg samples. Results showed a higher number of positive samples when nPCR was used, proving the suitability of nPCR to identify *B. bigemina*-infected ticks [42]. The same four pairs of primers were used in malaria-endemic regions of Colombia to detect babesiosis prevalence in humans, cattle and ticks that parasitized them, finding 18.5% of *Babesia*-infected ticks, out of which 73.3% were infected with *B. bigemina* and 16.7% with *B. bovis* [43]. The 18S ribosomal RNA gene has also been utilized as a target sequence to detect *Babesia bigemina* and *B. bovis* presences in the vector tick (Table 2). Guerrero et al. [44] used a PCR assay to detect the causal agent of bovine babesiosis outbreaks in Texas. To accomplish this, several tick strains taken from sick cattle were evaluated, along with Mexican and Brazilian tick strains. The assay sensitivity achieved with the PCR assay allowed detection of the equivalent of a single infected larva with both *Babesia* species, but only *B. bigemina*-positive *R. microplus* ticks were found.

A nPCR assay with primers specific to *msa-1* gene (Table 2) was developed to detect *B. bovis* DNA in *R. microplus* hemolymph samples with no kinetes detectable by light microscopy [35]. With the high analytical sensitivity reported for the nPCR (from 1 to 10 parasites), hemolymph samples collected from female ticks between 7- and 10-days post-repletion were analyzed. Out of a total of 62 females with no kinetes detectable by light microscopy, 32 were determined positive by nPCR with primers amplifying *msa-1*. Most importantly, it was found that larvae derived from replete females with exceptionally low levels of kinete infection, as demonstrated by light microscopy and nPCR, had infection rates from 22% up to 40% and transmitted *B*. *bovis* during transmission feeding experiments [35]. Thus, the differences in analytical sensitivity and specificity of the nPCR assay may account for the higher tick infection rates determined in that study. In addition, a real-time PCR assay performed on individual larvae from females having hemolymph samples with >10 kinetes per microscopic field showed levels ranging from 4.8 × 10^1^ to 1.2 × 10^5^ parasites per tick on day 3 of feeding in a recipient susceptible calf (Table 2).

A second study carried out utilizing spleen-intact, persistently infected calves with *B. bovis*, having lower parasite levels in peripheral blood, resulted in lower kinete levels in replete females and subsequently lower larval infection rates (0% to 20%), as estimated by using the nPCR assay [36]. Larvae tested by real-time PCR after 3 days of transmission feeding had parasite levels ranging from 2.4 × 10^2^ to 1.9 × 10^5^, values comparable to those found in the previous study, in which groups of larvae were derived from females harboring elevated levels of kinetes in their hemolymph [35]. The results showed that females fed on persistent carriers, despite low blood parasite levels, can acquire the parasite and pass it transovarially to larval offspring, which in turn are capable of transmitting *B. bovis* to a susceptible host [36].

In Egypt, prevalence determination of *B. bigemina* and *B. bovis* in *Rhipicephalus annulatus* (adult and nymph) ticks was achieved using a PCR assay [45]. To amplify the small subunit rDNA genes of each *Babesia* species, previously reported primers were implemented (Table 2). DNA samples used were obtained by pooling 2 to 4 nymphs, while adult ticks were utilized individually. Results showed 55% of *B. bovis*-infected ticks, 66% infected with *B. bigemina* and 12% infected with both parasite species. To confirm the authentication of the amplified PCR products, the amplicons were sequenced, and derived DNA sequences were submitted to a BLASTN homology search. *B. bovis* samples showed a high similarity with rDNA sequences of several Mexican isolates and one isolate from Israel, while *B. bigemina* samples had 100% sequence identity with the Spain isolate [45].

A different format of the PCR assay based in enzymatic digestion of PCR products to detect *Babesia bovis* and *B. bigemina* in its vector, the *Rh. microplus* tick, was carried out in Mexico [46]. The PCR-RFLP (Restriction Fragment Length Polymorphism) can identify both parasites at the same time, due to the amplification of a variable portion of the 18S rDNA gene, by using the Piro A/Piro B oligonucleotides designed to amplify different *Babesia* species [46]. After amplification, PCR products (400 bp) were digested with the restriction enzymes *Box I* and *Msp I*, that only recognize and cleave a specific site of *B. bigemina* and *B. bovis* sequences, with the recognition sequences 5′-GACNN↓NNGTC-3′ (*Box I*) and 5′-C↓CGG-3′ (*Msp I*), respectively (Table 2). With the PCR-RFLP assay, fragments of different size are produced after enzymatic digestion: two fragments of 250 and 150 bp are obtained for *B. bovis*, while two fragments of 290 and 110 bp are produced in *B. bigemina* samples. The PCR-RFLP assay is a precise and reliable technique, which can also be used for cattle monitoring during the acute phase of bovine babesiosis [47]. Disadvantages of the PCR-RFLP method include the requirement for specific endonucleases, the high purity and the amount of material collected to perform the reaction. Moreover, since PCR-RFLP consists of several steps, including an electrophoretic separation step, it is relatively time-consuming, and therefore the technique is not suitable for high-throughput analysis.

Another variant of the PCR assay is the quantitative Polymerase Chain Reaction (qPCR) technique, which was recently used in Brazil to study the host–*Babesia*–tick interaction. Through amplification of the mitochondrial cytochrome b gene of *B. bovis* and *B. bigemina* [48] (Table 2), the number of copies of each *Babesia* species present in larvae tick and blood samples was achieved, and although the number of base pairs expected for both species is the same (88 bp), the melting curves differ, which allowed the species differentiation. The qPCR assay had a high level of sensitivity; according to the Poisson distribution, at least three copies of the DNA fragment can be detected in the assay. Results obtained in this study displayed 100% of positive samples in either larvae or bovine blood, without significant differences between the number of copies found among *Babesia* species. Likely, in the case of tick samples, the results can also be a consequence of the larvae handling, because processed larvae (a pool of 100) were obtained from a pool of eggs oviposited by 10 adult engorged female ticks, thus increasing the chance of finding the minimum number of copies needed for amplification [49]. The same qPCR test was used for the detection of *B. bigemina* and *B. bovis* in *Rh. microplus* ticks fed on water buffalo and cattle. Tick samples were collected from calves and adult animals. The purpose of this study was to evaluate the capability for transmission between water buffalo–*Babesia*–ticks and prove the ability of buffalo to act as reservoir hosts. Despite ticks being found only in calves (80%) and adult buffalo not showing tick infestation, the reproductive performance of female ticks was similar in those obtained from water buffalo calves than those from cattle calves. Additionally, a similar *Babesia* positivity was found in the progeny of female ticks fed in cattle calves and water buffalo calves, with a higher presence of *B. bovis* in ticks as compared to *B. bigemina* [50].

Studies conducted on subolesin vaccination and release of tick larvae after subolesin knockdown by RNA interference (RNAi) have demonstrated to be effective for control of *Rhipicephalus (Boophilus) microplus* infestations in cattle [51]. By applying a qRT-PCR assay using primers that target the rDNA genes of *B. bigemina* (Table 2), the results showed that parasite infection levels were over 87% lower in ticks fed on subolesin-vaccinated cattle and after gene knockdown by RNAi when compared to control ticks [51]. Similarly, as determined by quantitative PCR (qPCR) of the 18S rDNA gene of *B. bigemina*, RNA interference studies in *R. annulatus* leading to knockdown of the Tick Receptor for Outer Surface Protein A (TROSPA) and serum amyloid A significantly reduced *B. bigemina* infection levels by 83% and 66% respectively, in *R. annulatus* when compared to control ticks. In *R. microplus*, knockdown of TROSPA and serum amyloid A also reduced pathogen infection levels by 70% and 86% respectively, while calreticulin knockdown resulted in 73% lower infection levels as compared with controls. However, subolesin knockdown did not affect *B. bigemina* infection levels in *R. annulatus* ticks [52].

Nested primer sets used to amplify a fragment of the *B. bovis rap1* gene [38] (Table 2) have also been utilized to determine tick infection rates in a parasite transmission experiment. Adult female ticks whose vitellogenin receptor gene had been RNA interference-silenced for expression in the ovary during tick feeding on *B. bovis*-infected cattle had reduced tick reproductive fitness [53]. An overall female infection rate of around 70% demonstrated that silencing the vitellogenin receptor did not affect *B. bovis* acquisition during tick feeding. However, *B. bovis* tick infection rates in larval progeny were 12% to 17% for non-silenced control groups, whereas there were no larvae infected with *B. bovis* from the vitellogenin receptor gene-silenced group, confirming that transovarial transmission of *B. bovis* to the offspring was diminished [53]. The Loop-Mediated Isothermal Amplification (LAMP) assay is another molecular technique that has been implemented to detect *B. bigemina* and *B. bovis* in infected cattle (Table 2). This method can amplify 10^9^ copies from template DNA and is less expensive and time-consuming than a conventional PCR assay. A LAMP assay developed in China was 1000-fold more sensitive than PCR for *B. bovis* and *B. bigemina* detection under analytical examination, when the PCR technique is performed with the same external primers as those used in the LAMP assay. Also, when used with field samples, a greater number of LAMP-positive samples were found as compared to the PCR assay [27]. However, when the same LAMP assay was compared with nPCR (primers set BV5650), only 90% of sensitivity for specific detection of *Babesia bovis* was displayed [54]. Perhaps the LAMP assay could be of great help in detecting *Babesia* parasites in the vector tick, but so far, no study has been reported.

#### 2.1.3. Other *Babesia* Species that Affect Cattle

A study was conducted to investigate the mechanisms that control *B. divergens* transmission in the tick vector *Ixodes ricinus*. By designing an artificial feeding technique that allowed infection of ticks with known numbers of parasites, the transstadial and transovarian transmission of *B. divergens* by *I. ricinus* was demonstrated [55]. Conventional PCR assays with 18S rRNA as the target gene (Table 2) performed on DNA extracted from tick salivary glands which identified the presence of *B. divergens* after molting of the artificially infected larvae and nymphs, as parasite DNA, could be amplified from all tested females and from all tested pools of nymphal salivary glands. In addition, PCR of the nymphs that molted from infected larvae fed on non-parasitized gerbils showed that parasite DNA was still present in salivary glands, indicating that the parasite persists from the larvae to the nymph (transstadial transmission). Similarly, positive PCR detection of parasite DNA on pools of eggs from adults infected by skin feeding demonstrated the transovarial transmission of *B. divergens*. PCR amplifications performed on hatched larvae 3 months later were also positive, indicating that the parasite DNA remained in the larvae. In all cases, sequencing of the PCR products showed that the recovered DNA was *B. divergens* [55]. In some cases, *Babesia*-infected ticks can be found on different invertebrate hosts other than those commonly parasitized, an important finding that was reported in Switzerland where *Ixodes ricinus* female ticks collected from goat, chamois and red deer were identified as *B. divergens*-positive. *B. major* was also found in *I. ricinus* female ticks derived from two red deer. *Babesia* sp. genotype EU1 was also found in *Ixodes ricinus* male ticks, suggesting the possibility of a transstadial transmission. Moreover, a new *Babesia* sp. denominated as genotype CH1, closely related to *B. odocoilei*, was reported in *I. ricinus* obtained from red deer. This finding was made possible by using a conventional PCR with generic and species-specific primers designed by the authors [56]. It was determined that better results would be found by using a more sensitive test such as the nPCR assay. This, coupled to detection of bovine *Babesias* in the wild ruminants sampled, could be a way forward for future research.

*Babesia orientalis* is another parasite commonly found in water buffalo in Asia [57], and a molecular technique designed for the detection of this parasite is the semi-nested PCR (Table 2). The assay has been successfully used in buffalo blood samples and *Rhipicephalus hemaphysaloides* tick samples, including the detection of the *Babesia* parasite in the tick progeny. The assay had an analytical sensitivity of 0.00000012% and did not show specificity when tested on other species of blood microorganisms that affect buffalo, such as bovine piroplasms, *Eperythrozoon wenyonii*, *Anaplasma marginale* and *Mycobacterium bovis*. The restriction endonuclease *SacII* was also implemented to confirm the nature of the amplicon obtained, with the production of two DNA fragments of 174 and 83 bp. In addition, PCR products were cloned and sequenced to confirm the identity with the 18S rRNA gene [57]. *B. orientalis* has also been identified in water buffalo by the LAMP assay, showing better results as compared to light microscopy and the semi-nested PCR assay mentioned above, though no studies were conducted in tick samples [58].

In 1981, a previously undescribed *Babesia* species was isolated from *Hyalomma marginatum rufipes* ticks collected from cattle in South Africa [59]. The piroplasms were morphologically like both *B. bovis* and *B. bigemina* but produced only mild clinical reactions even in splenectomized animals, and since the *Babesia* occurs at exceptionally low parasitemias, it was proposed as *B. occultans* [59]. Later, a study conducted in Tunisia reported the finding for the first time of *B. occultans* in *Hyalomma marginatum* ticks collected in northern Africa and described the use of the 18S rRNA gene (Table 2) as a target for the design of a species-specific probe for detecting *B. occultans* by Reverse Line Blot (RLB) hybridization [60].

A PCR assay was developed that allowed the identification of *Babesia ovata* in samples of *H. longicornis* and *I. ovatus* ticks. The assay was based on the beta-tubulin gene (*β-tubulin*) (Table 2), a component of microtubules in the cytoskeleton that is highly conserved among apicomplexan parasites, but with different sequence lengths. In the same study, a PCR test previously successfully used to detect *B. ovata* in cattle samples, based on apical membrane antigen-1 (AMA-1) as the target gene, was evaluated to detect *B. ovata*-positive ticks, showing inadequate results. None of the tick samples that were spiked with *B. ovata* DNA were amplified, probably due to non-specific binding of the primers with tick DNA. In contrast, the *β-tubulin* PCR assay displayed a better specificity and sensitivity to detect the protozoan parasite in tick samples [61]. Although *B. ovata* does not have a high economic impact in livestock, it is necessary to monitor its distribution in areas where other piroplasms are present due to the synergistic damage that they can cause.

### 2.2. Canine Babesiosis

Many different *Babesia* species have been reported as able to infect canids (Table 1), these are classified as large (2.5–5 µm) and small (1.0–2.5 µm) babesias [46,62,63]. *Babesia canis*, *B. rossi* and *B. vogeli* are known as the large babesias, while *B. gibsoni* and *B. conradae* are the smalls babesias [63,64]. Initially, the large babesias were identified as a unique species denominated as *B. canis*, but later they were reclassified based on their vector tick, distribution and pathogenicity: *Babesia canis canis*, *B. canis rossi* and *B. canis vogeli* [46,62,63], however, for the purpose of this review, they will be referred to as *B. canis*, *B. vogeli* and *B. rossi*. Besides being transmitted through a tick bite, *Babesia* transmission can occur during dog fighting, as some cases have been reported in the case of *B. gibsoni*, and more recently, with *B. canis*. In addition, a congenital transplacental transmission with *B. gibsoni* can occur [64,65]. The distribution of these *Babesia* spp. varies depending on the species: *B. gibsoni* and *B. vogeli* are the most widely distributed and are transmitted by *Haemaphysalis* spp. and *Rhipicephalus sanguineus* ticks, respectively [66]. *B. canis* is transmitted by *Dermacentor reticulatus* in Europe, *B. vogeli* is transmitted by *Rhipicephalus sanguineus* in tropical and subtropical countries and *B. rossi* is transmitted by *Hemaphysalis leachi* in South Africa [46,62,63,64].

#### Molecular Tools

Several formats have been developed to molecularly identify *Babesia* spp. (Table 2). Conventional PCR, the PCR-RFLP assay developed by Carret et al. [46] based on the primers PiroA and PiroB originally described by Olmeda et al. [67], as well as the semi-nested PCR assay developed by Birkenhauer et al. [68] have been used for the identification of the etiological agent of canine babesiosis in several parts of the world. In Italy, for example, by using the generic primers for the ssrRNA gene in the PCR assay, *B. canis* was identified in *Dermacentor marginatus* ticks infesting dogs [69]. In Mexico, the PCR-RFLP assay was performed by using the generic primers for the ssrRNA gene and the restriction enzymes *TaqI* and *HinfI* [70]. DNA sequencing of the amplicons obtained and BLAST analysis of the sequences revealed the identification of *B. vogeli* in *R. sanguineus* ticks. Similarly, a PCR-RFLP assay developed by Azmi et al. [71] based on the primers BJ1 and BN2 originally designed by Casati et al. [72] was used for the identification of *B. vogeli* in *Rh. sanguineus* ticks in Palestine [71]. The PCR-RFLP assay was performed using the generic primers BJ1 and BN2 for the ssrRNA gene and the restriction enzyme *XapI* (*ApoI*) (Table 2).

The semi-nested PCR assay developed by Birkenhauer et al. [68] was used with the generic primers 455–459/793–772 for the first amplification and the species-specific forward primer BCV (*B. vogeli*) combined with primer 793-772 for the secondary amplification [70]. The results obtained revealed the presence of *B. vogeli* in *Rh. sanguineus* ticks collected from clinically infected dogs by using both the PCR-RFLP and the semi-nested PCR methods. The comparison of the two methods showed that the semi-nested PCR assay had a higher specificity and analytical sensibility than the PCR-RFLP assay [70]. A multiplex PCR assay was recently reported in which *Ehrlichia canis*, *B. vogeli* and *B. gibsoni* DNA can simultaneously be detected in *R. sanguineous* ticks collected from infected dogs from India [73]. The assay was previously developed and used successfully for the detection of the same parasites in samples derived from sick dogs [74]. It was also found that *Hemaphysalis bispinosa* ticks were infected with *B. gibsoni* alone. The overall prevalence of infected *R. sanguineus* tick pools was 26.58%. Out of 7 *H. bispinosa* tick pools, 5 revealed the presence of *B. gibsoni*. However, transmission trials are still to be conducted to prove the vector of *B. gibsoni* in India.

Recently, a new conventional PCR assay has been developed for the simultaneous identification of *B. canis* and *B. vogeli* with the cytochrome c oxidase subunit 1 (*cox1*) as the target gene for PCR amplification [75] (Table 2). Parasites can be identified successfully in blood samples, having a minimum detection level of 3 × 10^−2^ infected erythrocytes/mL for *B. canis* and 2.1 × 10^−2^ for *B. vogeli.* Tick samples from *Rh. sanguineus* and *D. reticulatus* were also evaluated with the same primers (BFor/BvRev/BcRev), but only a 0.8% (1/128) tick infection rate was determined for *B. canis*. The amplicon was DNA-sequenced, finding a 99–100% similarity with *B. canis* sequences reported in GenBank [75]. Although the *B. canis* identification in *D. reticulatus* tick was achieved, further studies are required to verify the efficacy of this PCR assay and the vectorial capacity of this tick species. It has been well established that detection of DNA in a particular tick, without performing transmission trials, does not necessarily prove its competence as a vector for *Babesia* transmission [76]. Nonetheless, several studies have been conducted by using PCR methods to identify *B. canis*, *B. vogeli* and *Babesia gibsoni* in ticks. Some of them are based on PCR amplification of 18S rRNA gene fragments of *Babesia* spp., followed by sequencing the amplicons obtained (Table 2). In Great Britain for example, *B. gibsoni* DNA sequences were identified in *Ixodes ricinus*, a tick not previously identified as a competent vector for this parasite species [77]. Likewise, using a nPCR assay previously reported to amplify a fragment of the 18S ssrRNA gene of *Babesia* spp. [68] with the primers set (5-22F/1661R and 455-479F/793-722R) followed by DNA sequencing and subsequent phylogenetic analysis, the presence of *B. gibsoni* in nymphs of *Rhipicephalus sanguineus* ticks was identified for the first time in Asia [78]. *B. vogeli* has also been identified in nymphal and adult ticks, indicating a significant transstadial transmission in the lifecycle of this *Babesia* species and the possible existence of parasite competition for the tick vector, *Rhipicephalus sanguineus* [78,79]. Another PCR assay with the V4 variable region of 18S rRNA gene as a target, combined with Reverse Line Blot (RLB) hybridization and DNA sequencing of the amplified products [80], was used to identify *B. vogeli* in *R. sanguineus* ticks from Tunisia [81], *R. sanguineus* and *R. turanicus* from Israel [82], *R. sanguineus* and *R. haemaphysaloides* from India [83], and to define the local vector for canine babesiosis in Taiwan [84] (Table 2). In Taiwan, primers used for PCR amplification were RLB-F2/RLB-R2, and commercial oligonucleotides probes with *B. vogeli* and *B. gibsoni* as targets were used for RLB hybridization. *Hemaphysalis hystricis* females, males, nymphs and larval ticks showed a tick infection rate of 10.3%, 7.0%, 2.6% and 16.2% respectively, for *B. gibsoni*, suggesting a transovarian and transstadial transmission of *B. gibsoni* in *H. hystricis*. *B. vogeli* was also detected in female or male ticks and larval progeny of *R. sanguineus* ticks [84]. Similarly, the PCR-RLB hybridization assay using an improved protocol including a *B. canis*-specific probe [85] was utilized in a model to evaluate the prevention of transmission of *Babesia canis* by *Dermacentor reticulatus* ticks to dogs that were treated with a commercial acaricide formulation [86] (Table 2). Ticks sampled from an infestation batch were found to contain a 33.3% (17/51) *B. canis* infection rate, comprising 20% of 25 males and 46% of 26 females. It was demonstrated that 55 out of 122 (45.1%) ticks collected on day 6 post-infestation in untreated control animals were found to be infected with *B. canis*, while treatment of dogs with the combined formulation applied up to 28 days prior to infestation with *D. reticulatus* harboring *B. canis* successfully prevented development of canine babesiosis clinical signs.

*Babesia canis* has also been identified in ticks with the 18S rRNA gene as a target in a conventional PCR using primers BJ1 and BN2 originally designed by Casati et al. [72] or primers BcW-A and BcW-B [87], followed by DNA sequencing of amplicons. Studies with *Dermacentor reticulatus* adult ticks collected from healthy dogs in some European countries (Ukraine, Hungary, France, Italy, Belgium and Poland) and tested with these PCR assays (Table 2) showed overall prevalence rates of up to 20% [87,88,89,90]. More recently, a high-throughput real-time PCR-based array, using a microfluidic system, allowed for the identification of *B. canis* (16/1741) and *B. vogeli* (87/1741) in *Dermacentor reticulatus* ticks collected by blanket dragging in Great Britain and Germany/The Netherlands, respectively. In addition, *B. canis* results were confirmed by qPCR and conventional PCR followed by DNA sequencing of amplicons [91]. In addition, a nested PCR assay was developed and has been used to determine a 3.6% infection rate of *Babesia canis* in questing *D. reticulatus* ticks in Siberia [92]. The infection of unfed adult *D. reticulatus* ticks found in this study reflected the transstadial transmission of tickborne infectious agents. Using the same procedure, *Babesia* spp. were detected in 1.2% (26/2259) of *D. reticulatus* and in 9.5% (35/370) of *I. ricinus* ticks in Lithuania, whereas the overall prevalence of *Babesia* in *D. reticulatus* ticks from Latvia was 2.8% (5/181) [93].

Although some molecular techniques such as the LAMP assay [94] used for *Babesia canis canis* detection, and TickPath Layerplex (based on qPCR assay) designed for identification of different tick-borne pathogens that affect dogs (as *Babesia, Borrelia, Ehrlichia, Anaplasma* and *Rickettsia* genus) [95], have been designed to be implemented with invertebrate samples, their effectiveness for the identification of canine *Babesias* in ticks must still be tested.

### 2.3. Cervid Babesiosis

*Babesia capreoli, B. odocoilei* and *B. venatorum* are the causal agents of cervid babesiosis, and several wild and captative ungulates species have been identified as being infected with these parasites around the world, mainly in North America and Europe (Table 1). These *Babesia* species can be transmitted by Ixodid ticks such as *Ixodes scapularis*, *I. persulcatus* and *I. Ricinus.* While the majority of infected cervids do not present with symptoms and only a few clinical cases have been reported in Europe and recently in Canada, climate change and movements of migratory birds have resulted in babesiosis outbreaks in areas where they had not previously been reported. Additionally, *Babesia venatorum* is a parasite with high zoonotic potential in various Asian countries, hence the importance of its monitoring [96,97].

#### Molecular Tools

Conventional PCR was used in a recent study for detection of *B. venatorum* in *Ixodes persulcatus* ticks collected by flagging in Mongolia (Table 2). By using primers BJ1/BN2, amplification of a 411–452 bp fragment of the 18s RNA gene of *Babesia* spp. was achieved [98]. A total of 275 tick samples were evaluated and 3.3% of the ticks were *Babesia*-positive, out of which 4.1% (7/169) were female ticks and 2.2% (2/93) were male ticks. The specific identification of *B. venatorum* was made through the sequencing of the amplified fragments and the sequence analysis disclosed a 100% identity with the *B. venatorum* sequences already reported [98]. This PCR assay had been previously used for detection of different *Babesia* species in *Ixodes ricinus* ticks, but none of the samples gave a positive result for *B. venatorum* [72]. In another study carried out in Slovakia, using the same set of primers (BJ1/BN2) for the identification of *Babesia* spp. in *I. ricinus* ticks (larvae, nymph, adult) collected from free-living ungulates killed by hunting, fragments of 450 bp were obtained by PCR amplification and analyzed by DNA sequencing. *B. venatorum* was detected in three larvae samples derived from three different roe deer, and in one adult female tick derived from a fallow deer. The dead ungulates were also tested for *Babesia* spp., but none were positive, proving that the free-living ungulates can serve as carriers of infected ticks in Slovakia [99]. A current study carried out in Canada found the presence of *I. scapularis* ticks (larvae and nymphs) in several migratory birds sampled during spring and detected for the first time the presence of *Ba. odocoilei*-infected ticks in Ontario [100]. By using a semi-nested PCR assay, ticks collected from birds were tested for *Ba. odocoilei* by PCR amplification of piroplasm’s 18S rDNA partial sequence (Table 2). Results showed that 0.2% (2/1102) of sampled birds had *Ba. odocoilei*-positive larvae with a minimum infection rate of 1.2% (2/116) for *Ba. odocoilei*. The presence of *Ba. odocoilei*-positive ticks on migratory birds can represent a pathway for spreading babesiosis in immunologically naïve wild cervids in Canada. In the same study, *I. scapularis* ticks were collected from the ground by blanket dragging in sites inhabited by wild white-tailed deer. PCR analysis of all ticks sampled showed 1.9% (4/210) positivity for *Ba. odocoilei.* Furthermore, all positive samples were DNA-sequenced and compared with public databases, showing 99.5–100% identity with *Ba. odocoilei* isolates [100]. In another study conducted in France using the microfluidic real-time PCR (Table 2), *Babesia venatorum* was identified in salivary glands and midgut of unfed *I. ricinus* male ticks [101]. Adult males (30) were collected by flagging and the midgut and salivary glands were dissected out for DNA extraction. *B. venatorum* was identified in 13% of the salivary gland samples, whereas 7% of ticks were positive for *B. venatorum* in both salivary glands and midgut tissues. Additionally, these same organs were evaluated in unfed female ticks, but results were negative (0/30). The findings proved the capacity for *I. ricinus* male ticks to acquire the *Babesia* parasites, however, it is still necessary to confirm if the parasites present in salivary glands can infect cervids [101].

### 2.4. Equine Babesiosis

Equine babesiosis caused by *Babesia caballi*, considered as a true *Babesia* due to its obligate intraerythrocytic replication, is commonly transmitted by competent Ixodid ticks, such as *Dermacentor, Rhipicephalus* or *Hyalomma* ticks (Table 1). *B. caballi* has a worldwide distribution in tropical and subtropical regions, and although horses are the main vertebrate host, it can also be found in donkeys, mules and zebras. *B. caballi* is commonly found associated to *Theileria equi* (formerly *Babesia equi*) and both are responsible for the disease called Equine piroplasmosis [102,103].

#### Molecular Tools

Several molecular assays have been developed targeting one or multiple equine piroplasmosis parasites. Such assays include conventional PCR, nPCR, rtPCR, mPCR, RLB and LAMP (For a review see Tirosh-Levy et al. [104]). Most of these assays, however, have only been utilized on equine blood samples. As it is the case with other *Babesia* species, ticks and carrier horses can serve as a reservoir for the parasite, and the nPCR assay with species-specific primers has been implemented for the identification of *B. caballi* in ticks. In Mongolia, *B. caballi* was detected in adult *Dermacentor nuttalli* female ticks, and although ticks were collected from vegetation, this is the main species identified in Mongolian horses. The extracted DNA was subjected to a first PCR reaction with the primers BC48F1/BC48R3, and a second reaction (nPCR) was performed with the set BC48F11/BC48R31 for the specific amplification of 530 and 430 bp fragments, respectively (Table 2). Results showed a prevalence of 12.9% (7/54) positive ticks to *B. caballi* and sequencing of the nPCR amplified fragments exhibited an identical nucleotide sequence with the USA strain [105]. Recently, a new study carried out in northern Mexico found the presence of *B. caballi* in soft ticks by using the same nPCR assay. Fragments of 430 bp of the target BC48 gene that codes for a Merozoite Rhoptry Protein were successfully amplified, finding a molecular prevalence of 5.9% (3/51) in *Otobius megnini* ticks removed from the ears of sampled horses. Despite the findings obtained, future research is needed to verify the role and vector capacity of *O. megnini* ticks, such as the evaluation of salivary glands separately or by experimentation through controlled tick infestation [106]. Another PCR assay able to detect *Babesia caballi* and *Theileria equi* at the same time was used in horses and ixodid ticks (*Rhipicephalus bursa* and *Hyalomma* species) in Iran, but no DNA sample either from ticks or horses had *B. caballi*-positivity, although some horses showed antibodies against this piroplasm [107].

The Reverse Line Blot (RLB) hybridization technique has also been implemented to conduct prevalence studies on equine and tick populations infected with the piroplasmosis causal agents in Tunisia (Table 2). PCR amplification of a *Babesia* genus 18S rRNA gene was first performed, followed by hybridization with two probes previously reported [108]. The identification of *B. caballi* genotypes A and B in the equine blood samples and only *B. caballi* genotype B in *H. marginatum* ticks were achieved [109]. Other recently developed DNA-based diagnostic techniques are the loop-mediated isothermal amplification (LAMP) [110] and the rapid isothermal duplex real-time recombinase polymerase amplification (RPA) [25] assays for the diagnosis of equine piroplasmosis. However, the use of both the LAMP and the RPA assays for detection of the parasites in the vector ticks still needs to be explored.

### 2.5. Human Babesiosis

Human babesiosis is an emerging zoonotic disease usually caused by *Babesia microti* [17]. However, *B. crassa*-like, *B. divergens*, *B. divergens*-like, *B. duncani* and *B. venatorum* can also be primary agents of human babesiosis [30,111]. *B. microti* is the most common cause of human babesiosis in the USA, although sporadic infections with *B. divergens*-like and *B. duncani* have also been reported [30,111] (Table 1). *Ixodes scapularis* ticks are the predominant vector for *B. microti* and white-footed mice are the natural *Babesia* reservoir [30,111]. Moreover, *I. scapularis* can infest white-tailed deer, serving as blood supplier, but deer do not acquire the protozoan parasite. On the other hand, *B. duncani* is transmitted by *Dermacentor albipictus* and *Babesia divergens*-like is transmitted by *Ixodes* spp. ticks. While human babesiosis in Europe is mainly caused by *B. divergens* and occasionally by *B. microti* and *B. venatorum*, *Ixodes ricinus* ticks are the main vector of these three species. Whilst, in China, *B. venatorum* and *B. crassa*-like are the main causal agents of babesiosis and are transmitted by *I. persulcatus* ticks. Isolated cases of human babesiosis have been reported in Australia, Cuba, Egypt, India, Mexico, South America and South Africa [9,112].

#### 2.5.1. Molecular Tools

PCR assays have also been used for the identification of *Babesia* parasites in ticks obtained from infested humans. Such is the case of a study conducted in The Netherlands where the conventional PCR, PCR-RLB and qPCR assays (Table 2) have been used for *Babesia* detection in *Ixodes ricinus* ticks obtained from several patients with exposure to a tick-bite [113]. *Babesia* spp. was detected in 3.55% (11/314) of the tick samples obtained from 293 patients, of which 278 patients consulted their physician for a tick bite, and fifteen patients consulted their physician with an erythema migrans. *B. microti-* (6/314), *B. venatorum-* (4/314) and *B. divergens* (1/314)-positive tick samples were found by using the PCR-RLB assay. *Babesia*-positive tick findings were not enough to infer the probability of *Babesia* infection in The Netherlands but helped to focus on the possibility of detecting human babesiosis outbreaks in that country [113].

On the other hand, a new human babesiosis case has been reported in South Korea, where *Babesia motasi* was detected in humans for the first time [114]. This hemoparasite is commonly found in sheep and is transmitted by *Hemaphysalis longicornis* ticks. A total of 597 ticks were collected near the patient’s residence area. Several conventional PCR assays were carried out to detect the causal agent of babesiosis and to identify possible *Babesia*-infected ticks. The primers used for the identification of *Babesia* spp. were based on the target genes 18S rRNA, Cytochrome b (*COB*) and Cytochrome c oxidase subunit III (*COX-3*). The species-specific primers for the detection of *B. microti* (*β*-tubulin) and *B. divergens* (18S rRNA) were also used (Table 2). Only three *H. longicornis* ticks showed positivity for *Babesia* spp., by amplification of the 18S rRNA and *CoB*/*Cox-3* genes, whereas one tick was found positive for *B. microti.* Additionally, amplicons from the positive samples were sequenced, multiply aligned and a phylogenetic tree was constructed with the results. Although *B. motasi* was found in an immunosuppressed human, which highlights its possible zoonotic capacity, more studies are necessary to prove that this parasite represents a medical threat [114].

The reverse transcriptase PCR assay with 18S rRNA of *Babesia microti* as the target gene (Table 2) has allowed for the development of a tick surveillance method for monitoring human babesiosis in the USA [115]. To accomplish that, *B. microti* infection prevalence in *Ixodes scapularis* nymphs was determined in several endemic and non-endemic human babesiosis areas, and a regression model was used to associate it with the incidence rates for human babesiosis [115]. *Babesia duncani* is another zoonotic species that has presumably been identified in the United States since 1968. Although several cases have been identified since then, the tick vector and the natural host had not been confirmed in the western North America until 2019, when monitored larvae, nymphs and adults of *Dermacentor albipictus*, previously suspected as the vector tick, were found positive [116]. The *β-tubulin* gene from *B. duncani* was amplified by nPCR assay with the F34/R323 and BtubFn/BtubRn primers (Table 2) and determined to have an analytical detection sensitivity of 1 copy of template DNA [116]. Presence of *B. duncani* was confirmed in larvae and adult ticks collected, and a high prevalence was present in tested mule deer. Even though the DNA in adult ticks was correctly amplified by the nPCR, the amplicons generated could not be sequenced and a conventional PCR with the 18S RNA gene was performed for subsequent sequencing and confirmation of the positive samples. Finally, the amplicons from the *β-tubulin* gene obtained from the larval ticks were sequenced and bioinformatically analyzed, confirming high identity with the WA1 strain of *B. duncani*, suggesting a transovarian tick transmission [116]. On the other hand, *Babesia venatorum* and *Babesia microti* were recently identified in *I. ricinus* ticks recollected from dogs in Denmark, and although only one human babesiosis imported case has been reported in this country, the recent findings should be a starting point for future research on babesiosis [117].

#### 2.5.2. Biological Tools

A biological method based on the isolation of *Babesia* parasites from *Ixodes persulcatus* ticks and its subsequent parasite cultivation was carried out in China to identify and prove the vectorial capacity of *I. persulcatus* ticks [118]. Female and male ticks collected by the flag-drag method were allowed to feed on pathogen-free Severe Combined ImmunoDeficiency (SCID) mice. Only two out of twenty mice infested with *I. persulcatus* female ticks showed *Babesia* forms by blood smear microscopic examination, whereas none of the mice infested with male ticks showed *Babesia* parasites. Blood samples from *Babesia*-positive mice were used to establish the in vitro culture of the parasites by the Microaerophilic Stationary Phase System (MASP) method, achieving the isolation of *Babesia* spp. By amplifying a fragment of the nss-rRNA gene by PCR, using the Piro A/Piro B primers followed by DNA sequencing and phylogenetic analysis, the parasites were later identified as *Babesia microti*. A DNA sequence similarity greater than 99% to *B. microti* isolates reported as zoonotic in Asia was found. While human babesiosis cases had not been officially reported in China before this study was carried out, it is probable that human babesiosis cases have been misdiagnosed due to the presence of either other tick-transmitted pathogens or a lack of species-specific detection methods [118]. Recently, larvae and nymphs of *R. hemaphysaloides* ticks that fed on laboratory mice infected with *B. microti* were detected as positive by PCR at 4 weeks post-molting [119]. More importantly, it was experimentally demonstrated that *B. microti* can be transmitted artificially by *R. hemaphysaloides* to Bagg Albino (BALB)/c and Non Obese Diabetic (NOD)/SCID mice when infested with nymphs molting from larvae that ingested the blood of infected mice, suggesting that this tick might be a potential vector of human babesiosis in southern China [119].

These and other types of biological tools have also been previously used in human babesiosis diagnostics to experimentally demonstrate the vectorial capacity of the tick *Ixodes ricinus* to transtadially or trans-ovarially transmit other important zoonotic *Babesia* sp. For example, to reproduce part of the parasite cycle that occurs in the tick vector, *Ixodes Ricinus*, an in vitro animal skin feeding technique in which larvae or nymphs feed on blood containing in vitro-cultivated *B. divergens*, was developed by Bonet et al. [55,120]. It was shown that parasite DNA was detected in all samples of salivary glands of nymphs and adults that had fed on parasitized blood as larvae and nymphs respectively, indicating acquisition as well as a transtadial persistence of *B. divergens*. Moreover, PCR analysis performed on eggs and larvae produced by females that had fed on parasitized blood demonstrated the existence of a transovarial transmission of the *Babesia* parasite in the *I. ricinus* ticks [55,120].

Later, another study using the Microaerophilic System for in vitro cultivation of *Babesia* sp. showed that *I. ricinus* ticks are competent vectors for *Babesia* sp. EU1 [121]. By collecting engorged female ticks that fed on *Babesia*-infected cattle and extracting the salivary glands out of the infected ticks, it was demonstrated that not only can these ticks carry *Babesia* sp. EU1 DNA, but more importantly, they enable these parasites to complete their lifecycle up to the production of infectious sporozoites, as the experimental set-up using *Babesia* in vitro culture conditions allowed the direct invasion of erythrocytes by *Babesia* sp. EU1 sporozoites present in the salivary gland extracts [121].

It has previously been well-established that there is no doubt that effective in vitro feeding systems for Ixodid ticks of medical and veterinary importance have major benefits [55,120,121,122]. Although feeding ticks on live experimental animals seems apparently simple, it is not practical, as not all the biological models currently available are ethically acceptable. Thus, several methods have been developed to feed and infect ticks artificially [120,121,122], and in the case of zoonotic *Babesia*, the membrane feeding technique mimics reality more closely than other techniques [120]. Each technique has advantages and disadvantages, in all cases, however, infecting ticks under controlled conditions allows for testing biological questions such as studying the pathogen development inside their vectors [120,121,122], to identify the mode of transmission for a particular *Babesia* species within a competent tick vector, and eventually, evaluate the effect of pharmaceutical drugs or immunological reagents on the parasite development within the tick [118,119,120,121,122]. While especially useful, these techniques tend to present with long and difficult set-up periods, giving sometimes unpredictable results, and require efforts to standardize and simplify the laboratory protocols [120,121,122].

### 2.6. Ovine Babesiosis

*Babesia ovis* is the principal etiological agent of clinical babesiosis developed in goats and sheep [123]. Even though the parasite can be transmitted by *Rhipicephalus bursa*, *R. sanguineus* and *R. turanicus* (Table 1), transovarian transmission only occurs in *R. bursa* [124]. It has been suggested that unfed adult *R. bursa* ticks (either males or females) is the principal stage able to transmit the parasite in adequate quantities or with a better capacity of infectiousness for the development of babesiosis in lambs, and to a lesser extent, the nymphal tick stage [125]. *B. motasi* is another species that affects small ruminants and is transmitted by *Hemaphysalis punctata.* It has been suggested that two subspecies with different pathogenicity levels and distribution exist: *B. crassa* can also be found in sheep and goats but do not cause severe disease [8]. A novel ovine *Babesia* has been found in China denominated as *Babesia* sp. Xinjiang. Larval and adult *H. a. anatolicum* ticks can transmit the parasite to the vertebrate host. *Babesia* sp. Xinjiang has a low level of pathogenicity, principally in splenectomized sheep, but can elucidate serious symptoms when it infects immunosuppressed animals [126]. Recently, a new *Babesia* species parasitizing sheep and goats has been reported in Turkey. The *Babesia* sp. is genetically different from other known ovine species, and has a close similarity with *B. odocoilei*, *Babesia* sp. EU1 and *B. divergens.* Although no studies were conducted on the possible vector tick, it is likely to be transmitted by *R. bursa* or *R. turanicus* [127]. Ovine babesiosis could be found in the Mediterranean basin, Africa, Asia and Europe, where the vector ticks are commonly present. Diseased ruminants can present a severe anemia with a Packed Cell Volume (PCV) decrease around 30–40%, as well as fever, icterus and hemoglobinuria [8,123].

#### 2.6.1. Microscopy Tools

The microscopical examination of hemolymph and egg tick samples has been of great help in understanding the *B. ovis*–tick-vector–sheep interaction. Yeruham et al. [128] demonstrated that there is a correlation between the parasitemia level present in lambs with the number of kinetes present in hemolymph and eggs derived from adult female *R. bursa* ticks: the higher the level of parasitemia in sheep, the greater the number of kinetes present in hemolymph. The study carried out utilized two lambs infected with *B. ovis* presenting with different levels of parasitemia (10% and 0.3%). Microscopic examination of Giemsa-stained hemolymph and egg smears identified between 1156 ± 660.13 and 9.5 ± 12.02 kinetes in 0.5 μL of hemolymph obtained from engorged ticks derived from lambs with high and low parasitemia, respectively. Similarly, a greater number of kinetes were found in eggs oviposited by ticks fed on a lamb with high parasitemia. The level of parasitemia also had an effect on the number of oviposited eggs and the oviposition time, as the quantity of eggs laid was lower and in less time in ticks that fed on blood with higher parasitemia, but no affectation was perceived on the percent and time of hatchability [128]. In an epidemiological study conducted in Iran [129], the microscopical detection method was used to determine the ovine babesiosis prevalence in sheep and in the potential tick-vector. Giemsa-stained smears of hemolymph and egg samples from *R. sanguineous* and *H. marginatum* ticks were examined to search for kinetes, although only a low percentage of tick samples were found positive as compared to the *Babesia ovis* and *B. motasi* infection rate (28%) present in sheep. The low prevalence of ovine *Babesia* in the tick samples could be related to the low parasitemia present in the animals sampled, according to the correlation found by Yeruham et al. [128].

#### 2.6.2. Molecular Tools

*Babesia ovis* can be detected in vertebrate and invertebrate hosts by PCR amplification of the small subunit ribosomal RNA gene (Table 2). Prevalence studies of *B. ovis* in small ruminants and *R. bursa* ticks in Iran was carried out by PCR using Bbo-F/Bbo-R primers [130]. The prevalence fluctuated between 13% and 20% with a higher presence of *B. ovis*-infected ticks in goats than in sheep. A PCR-RFLP assay with the *Hph*I restriction enzyme was implemented to detect *B. ovis* in *R. bursa* (Table 2). Only a single PCR-RFLP profile was identified, indicating the existence of a single strain of *B. ovis* in the analyzed samples [124]. Another study with the 18S rDNA gene as a target reported the presence of *B. ovis* in *R. bursa* and *R. turanicus* ticks collected on sheep and goats in Argelia [131]. Although *Babesia* spp. identification was initially carried out by a qPCR assay using the primers set (Bab_18s_F/Bab_18s_R) and the probe (Bab_18s_P), *B. ovis* positivity was confirmed by a conventional PCR [132] followed by sequencing.

Another PCR assay based on the gene that codes for the *B. ovis* Surface Protein D (BoSPD) (Table 2) was evaluated on tick tissues and ovine blood samples from animals experimentally and naturally infected with *B. ovis* [133]. Ticks were infected by allowing larvae and adults to feed, until detachment, on a splenectomized lamb infected with *B. ovis*. For DNA extraction and PCR detection, 10 adult detached males were carefully dissected to remove their salivary glands and gut and pooled together. A positive BoSPD PCR signal was observed in both the gut and the salivary gland extracts. In addition, successful detection of *B. ovis* in field samples of ovine blood and *R. bursa* ticks in a situation where sub-clinical infection occurs and the animals do not show clinical signs demonstrated the usefulness of the BoSPD PCR in studies of the long-term effect of *Babesia* infection on sheep and ticks [133]. The PCR assay was utilized to identify the *R. bursa* tick stage responsible for *B. ovis* transmission in small ruminants. Acquisition and transmission studies were conducted on intact and splenectomized lambs infested with larvae, nymphs, female and male adult ticks that fed on lambs inoculated with *B. ovis*-infected erythrocytes. Monitoring the clinical response to the infection by serological and molecular assays and the presence of parasites in tick samples by PCR, the results showed that about 25–35 adult (males or females) *R. bursa*-infected ticks were sufficient to transmit the parasites and cause severe babesiosis in lambs, while 0.5 g larvae or 200 nymphs were insufficient to cause clinical babesiosis [125]. Later, a real-time PCR analysis for quantitative evaluation of persistent parasitemia in infected lambs was applied to *R. bursa* ticks from naturally infected adult sheep (Table 2). Results obtained using the quantitative PCR with BoSPD primers showed that less than 10 target copies could be detected per reaction, an analytical sensitivity greater than what was obtained by conventional PCR [134]. Then, the BoSPD qPCR was applied to DNA extracts of pools from 10 individual ticks. The efficient quantitative evaluation of *B. ovis* in *R. bursa* salivary glands during the adult post-molting and pre-feeding stage demonstrated the usefulness of a *B. ovis*-specific qPCR procedure, showing that the relative parasite load increases rapidly upon completion of the post-molting stage. This finding suggests that parasite multiplication is coordinated with the completion of the tick post-molting stage, towards initiation of the feeding stage [134].

As with other *Babesia* species, the LAMP assay has been developed and used for the identification of *Babesia* sp. BQ1 (Lintan) and *Babesia* sp. Xinjiang-200 (piroplasms affecting small ruminants such as goats and sheep in northern China), with an analytical sensitivity of 0.02 and 0.2 pg respectively, a higher sensitivity than that achieved with the nPCR assay (2.0 pg) [135]. However, no studies have been conducted with tick-vector samples.

## 3. Discussion and Conclusions

*Babesia* spp. detection in vector ticks remains a challenge today. Despite that a number of methods have been developed and are currently available to be used for that purpose, most of them, especially those based in the molecular detection of the parasite, were initially designed for the identification of the *babesia* parasite in the vertebrate host and have been later on adapted for use with DNA samples derived from ticks. The ineffectiveness of the tests when working with tick samples has been reported in some instances: such is the case for *Babesia ovata* detection in *H. longicornis* and *I. ovatus*. A conventional PCR assay with AMA-1 as the target gene did not amplify the parasite DNA due to the primers’ binding un-specificity with the DNA of ticks, even though the PCR had previously been used successfully with blood samples [61]. For this reason, it is imperative to perform in silico trials with the nucleotide sequences of both organisms, the tick vector and the *Babesia* parasite, to identify possible mispairs and avoid false positives and negatives. Another important problem to be solved is the inhibition of the PCR reactions caused by components present in the processed samples, although in general, this problem has been solved through a pre-processing step including washing and sterilization of the whole tick samples. Several authors advise the use solely of dissected tick tissues, such as the salivary glands, ovaries and the midgut, where *Babesia* spp. parasites are commonly present [29,101,133,134]. Despite this, the advice has not been accurately described and followed by most of the authors cited here, most probably due to the excessive consumption of time necessary for tick processing and the need for qualified operators to carry out the task.

Most of the techniques employed for detection of the different developmental stages of the various *Babesia* species in the vector ticks are based on the molecular identification of parasite DNA. PCR-based methods such as the conventional PCR, semi-nested PCR and nPCR are the most frequently used assays for *Babesia* sp. detection, and for the establishment of tick infection rates. Through fragment amplification of a nucleotide sequence from a target gene, it is possible to identify the presence of the *Babesia* parasites in tissues derived from ticks sampled for epidemiological studies. The 18S rRNA gene is the target gene most widely used for the molecular identification and phylogenetic analysis of parasites that belong to the phylum Apicomplexa, including the genus *Babesia* due to its high conservation and expression [136,137]. Recently, it was reported that in *Babesia microti*, the rRNA gene is over 1000-fold more abundant than coding genes [138]. Although several studies referenced in this article used the 18S rRNA gene to identify *Babesia* in tick samples by PCR assays, this was generally achieved by the amplification of a species-specific region or by the amplification of a conserved common region among *Babesia* species, followed by the posterior sequencing of the amplified fragments and the analysis of the sequences obtained in both cases. Another target gene used successfully for detection of *Babesia* species is the *β-tubulin* gene, which is also strongly conserved among Apicomplexa parasites. Depending on the *Babesia* species, the gene contains one or more introns, that allows the differentiation of parasite species based on the nucleotide sequence and the length of the amplified sequence [139]. Detection methods based in the morphological identification of *Babesia* parasites using microscopy tools are currently less employed, primarily due to the need for qualified operators, the time consumed during processing and analysis and the presence of *Babesia* parasites in low quantities in the tick hemolymph. In the field studies with ticks cited here, the differentiation between *Babesia* species present in *Rhipicephalus microplus* ticks that affect the Brazilian cattle was not possible, therefore the samples were only identified as *Babesia* spp. kinetes [34,37]. Nonetheless, the microscopic analyses of *Babesia* stages in the tick vector are still essential while performing tick transmission studies and are an ideal complement for the molecular tools available, especially those that provide a quantitative result that can be used to estimate the intensity of *Babesia* infection or parasite loads within the tick vector.

Moreover, the molecular identification of *Babesia* species in the different ticks and mammal hosts most commonly infested does not necessarily mean that these play an important role in the epidemiology and spread of the *Babesia* parasites; for such assertion, further transmission experiments, accompanied with serological and microscopical methods as well as clinical examination, are needed [140], such as the case of *Babesia caballi*, recently found in soft *Otobius megnini* ticks [106]. On the other hand, the finding of *Babesia* spp. in other possible vectorial ticks and in new geographic regions, as well as the variation in the abundance of ticks, are important factors to investigate due to existing climate changes around the world. Lack of previous exposure to ticks, and consequently to *Babesia* parasites, could cause babesiosis outbreaks in susceptible mammals [4,141], hence the importance of tick monitoring to prevent possible outbreaks and to identify the vector ticks for the *Babesia* species present in a certain region, as well as to discover the causal agents of diseases in areas where other parasites are commonly present, particularly those with a zoonotic potential. In this sense and considering that increasing human travel, animal transport and environmental changes have been important elements responsible for the emergence and/or spread of numerous tick-borne pathogens in Europe [2], more effective tick-based surveillance is essential for monitoring human and/or animal disease emergence [115]. To accomplish this, a new investigative tool which performs high-throughput testing of a wider panel of tick-borne pathogens was developed [142]. The molecular tool utilizes a microfluidic system that can perform parallel real-time PCRs using multiple primers/probes sets able to perform high-throughput detection of tick-borne pathogens with 96 × 96 chips or 48 × 48 chips, resulting in either 9216 or 2304 individual reactions. In a single experiment, 94 ticks or pools of ticks (questing *I. ricinus* nymphs collected using the flagging technique) can be tested for the presence of 25 bacteria (8 *Borrelia* sp, 5 *Anaplasma* sp, 4 *Ehrlichia* sp, 4 *Rickettsia* sp, 2 *Bartonella* sp, 1 *Francisella* sp, 1 *Coxiella* sp) and 12 parasites, including *B. divergens*, *B. caballi*, *B. canis*, *B. vogeli*, *B. venatorum*, *B. microti*, *B. bovis*, *B. bigemina*, *B. major* and *B. ovis*, and 2 *Theileria* sp. [142]. The technique can, in the future, be utilized for large-scale studies utilizing the unique ability to simultaneously analyze large numbers of tick samples and multiple target pathogens, particularly in vector surveillance studies for monitoring emergence of human and animal diseases. As ticks may also be co-infected with several pathogens, with a subsequent high likelihood of co-transmission to humans or animals, the technique can be amenable to perform large-scale epidemiological studies to reveal the pathogen co-infection rates in ticks, which can possibly be co-transmitted to humans or animals. The high-throughput technique can also be utilized to estimate prevalence rates of symbionts co-existing with pathogens, which can be useful to study the potential effects on pathogen transmission and vector competence [143]. Recently, the microfluidic real-time PCR system was utilized to determine tick-borne pathogens of zoonotic importance in ticks collected from dogs in three different agro-ecological zones of Punjab, Pakistan [144], tick-borne pathogens present in ticks collected on different Corsican animal hosts, focusing on the main pathogens of medical and veterinary importance known in the Mediterranean area [145], tick-borne microorganisms of small ruminants from five districts of the Federally Administered Tribal Area of Pakistan [146], and to investigate the tick-borne microorganisms in ticks collected from cattle and water buffalo out of different agroecological zones in Pakistan [147] and identify the main bacteria and protozoans potentially transmitted by ticks in the Caribbean [148]. The results of the various studies conducted recently using the microfluidic real-time PCR system have provided an extremely useful diagnostic tool for direct detection of up to 50 bacterial and parasitic species within a single experiment or surveillance study, helping in establishing the distribution patterns and the control of tick-borne pathogens of livestock, of companion animals and of those with zoonotic potential. Thus, the microfluidic real-time PCR diagnostic system can supply broader capacities for the surveillance of potentially pathogenic microorganisms by targeting the main bacterial and protozoan genera involved in human and animal vector-borne diseases [142,143,144,145,146,147,148]. Most recently, a newly developed approach has been established and utilized in Pakistan to elucidate the composition of the piroplasm populations in cattle and buffalo [149]. By using a PCR-based Next Generation Sequencing (NGS)-informatic platform system with the V4 hypervariable region of 18S small subunit rRNA gene from piroplasms as a target gene, the study revealed piroplasms in 68.9% of the samples analyzed, with higher overall occurrence for *T. annulata* (65.8%), followed by *B. bovis* (7.1%), *B. bigemina* (4.4%) and *T. orientalis* (0.5%), demonstrating the identification of mixed infections and the discovery of *B. occultans* in Pakistan. It was proposed that variations in composition of piroplasm populations in bovines and buffalo as well as in the different geographical regions studied is most likely associated with differing prevalence’s of suitable tick-vectors. It is envisioned, therefore, that this approach could now be proposed as a powerful tool to be applied in conducting studies on the abundance and diversity of pathogens in the tick-vector populations infecting livestock. Due to its high analytical sensitivity and specificity, the PCR-coupled NGS-informatic approach system will allow to explore temporal and spatial changes in piroplasm compositions in both livestock animals and associated tick vectors, and it could also assist in assessing the effectiveness of anti-pathogen vaccines or to assess the vector competence for piroplasm infections.

## Figures and Tables

**Table 1:** Overview of *Babesia* species detected in Ixodidae ticks referenced in this study and the detection methods used.

*Babesia* Species	Main Tick Vectors	Mammal Host	Geographical Distribution	Detection Methods	Tick Stage Evaluated	References
*B. bigemina*	*Rhipicephalus microplus*, *Rh. decoloratus*, *Rh. annulatus*, *Rh. geigyi, * *Rh. evertsi*	Cattle, Water buffalo	Africa, America, Australia	Microscopy, nPCR, PCR, PCR-RFLP, qPCR	Adult, nymphs, larvae, eggs. Hemolymph and eggs	[34,37,40,41,42,43,45,47,48,49,50,51,52]
*B. bovis*	*Rhipicephalus microplus*, *Rh. decoloratus*, *Rh. annulatus*, *Rh. geigyi*	Cattle, Water buffalo	Africa, America, Australia	Microscopy, nPCR, PCR, PCR-RFLP, qPCR	Adult, nymphs, larvae, eggs. Hemolymph and eggs	[34,35,36,37,40,41,43,45,47,48,49,50,53]
*B. divergens*	*Ixodes ricinus, I. persulcatus*	Cattle	North West Europe, Great Britain, Ireland, and Spain	PCR, PCR-RLB hybridization	Adult, nymphs, (Salivary glands) larvae, eggs.	[55,113]
*B. occultans*	*Hyalomma marginatum*	Cattle	Africa	RLB hybridization *B. occultans-*specific probe	Adult	[60]
*B. ovata*	*Haemaphysalis longicornis, Ixodes ovatus*	Cattle	China, Japan, Korea, Mongolia, Thailand	PCR	ND	[61]
*B. canis*	*Dermacentor reticulatus*	Dogs	Asia and Europe	PCR, real-time PCR-based assay, qPCR, PCR-RLB hybridization, *B. canis*-specific probe	Adult	[69,75,85,86,87,88,89,90,91,93]
*B. vogeli*	*Haemaphysalis* spp., *Rhipicephalus sanguineus, * *Rh. turanicus, Rh. haemaphysaloides*	Dogs	Africa, Asia, Australia, North and South America, Europe	PCR-RFLP, semi-nested PCR, PCR, nPCR, PCR-RLB hybridization, real-time PCR-based assay, Multiplex PCR	Adult, nymphs, larvae, males, and unfed females	[70,71,73,74,78,79,81,82,83,84,91]
*B. gibsoni*	*Haemaphysalis* spp., *Rh. sanguineus, Rh. turanicus, Ixodes ricinus*	Dogs	Africa, America, Asia, Australia, Europe	PCR, nPCR, PCR-RLB hybridization, Multiplex PCR	Adult, nymphs, larvae	[73,74,77,78,84]
*B. venatorum*	*Ixodes ricinus, I. persulcatus*	Dogs, Roe deer, Red deer, Fallow deer, Moose, White-tailed deer, European reindeer	Canada, China, Europe, Mongolia and USA	PCR, microfluidic real-time PCR, PCR-RLB hybridization	Adult, larvae	[77,93,98,99,101,113]
*B. odocoilei*	*Ixodes scapularis*	White-tailed deer	Canada, USA	semi-nested PCR	Adult, Larvae	[100]
*B. caballi*	*Dermacentor* spp., *D. nuttalli, D. nitens, Hyalomma marginatus*, *H. truncatum*, *Otobius megnini*	Horses, Donkeys, Mules and Zebras	Africa, America, Asia, Europe	nPCR	Adult	[105,106]
*B. microti*	*Ixodes scapularis, I. ricinus*	White-footed mouse, Humans	United States, Europe	PCR-RLB hybridization, nPCR, RT-PCR, in vitro culture-PCR	Adult females, Nymphs	[113,114,115,118]
*B. motasi*	*Rhipicephalus bursa, Haemaphysalis longicornis, H. punctata*,	Goats, Sheep	Korea	Microscopy, PCR	ND	[114,129]
*B. duncani*	*Dermacentor albipictus*	Mule deer	United States	nPCR, PCR	Adult, larvae	[116]
*B. ovis*	*Rhipicephalus bursa*	Goats, Sheep	Iran	Microscopy, PCR-RLB, qPCR, PCR	Adult, eggs, larvae	[124,125,128,129,131,133,134]

ND: tick stage was not defined.

**Table 2:** PCR oligonucleotide sequences used for detection of various *Babesia* species in the tick-vector reported in different studies. The identity of *Babesia* parasites amplified with non-specific primers was confirmed through sequence analysis.

*Babesia* Species Detected	PCR Format	Target Gene or Region	Primers Name	Product Size	Primer Sequence	References
*B. bigemina* and *B. bovis*	nPCR	*SpeI*-*AvaI* *rap-1*	BiIA/BiIB BiIAN/BiIBN BoF/BoR BoFN/BoRN	278 bp 170 bp 356 bp 291 bp	5′-CATCTAATTTCTCTCCATACCCCTCC-3′ 5′-CCTCGGCTTCAACTCTGATGCCAAAG-3′ 5′-CGCAAGCCCAGCACGCCCCGGTGC-3′ 5′-CCGACCTGGATAGGCTGTGTGATG-3′ 5′-CACGAGGAAGGAACTACCGATGTTGA-3′ 5′ CCAAGGAGCTTCAACGTACGAGGTCA 3′ 5′-TCAACAAGGTACTCTATATGGCTACC-3′ 5′-CTACCGAGCAGAACCTTCTTCACCAT-3′	[38,40,41,42,43]
*B. bovis*	nPCR	*msa-1*	external forward external reverse internal forward internal reverse	ND 212 bp	5′-TTCGACCAGACCAAATTGT-3′ 5′-CGCATCAAAAGA CTCAACA-3′ 5′-GCCCTGATCTATTTAATGCA-3′ 5′-CCCCGTATAAACATGCTTC-3′	[35,36]
*B. bovis*	qRT-PCR	*msa-1*	Forward/reverse Probe	150 bp	5′-GATGCGTTTGCACATGCTAAG-3′ 5′-TGAGAGCACCGAAGTACCCG-3′ 5′-CACGCTCAAGTAGGAAATTTTGTTAAACCTGGA-3′	[35,36]
*B. bovis*	nPCR	*rap-1*	BoF/BoR BoFN/BoRN	354 bp 291 bp	5′-CACGAGGAAGGAACTACCGATGTTGA-3′ 5′ CCAAGGAGCTTCAACGTACGAGGTCA 3′ 5′-TCAACAAGGTACTCTATATGGCTACC-3′ 5′-CTACCGAGCAGAACCTTCTTCACCAT-3′	[53]
*B. bigemina* and *B. bovis*	nPCR	18S rRNA	KB-16/KB-17 KB-18/KB-19 KB-24/KB-25	ND 262 bp 217 bp	5′-CATCAGCTTGACGGTAGGG-3′ 5′-GTCCTTGGCAAATGCTTTC-3′ 5′-GATGTACAACCTCACCAGAGTACC-3′ 5′-CAACAAAATAGAACCAAGGTCCTAC-3′ 5′-GGGGGCGACCTTCAC-3′ 5′-CTCAATTATACAGGCGAAAC-3′	[44]
*B. bigemina* and *B. bovis*	PCR	ssrDNA	A/B C/B	118 bp 225 bp	5′-TGTCCTCGTTTGCTTCTTAGAGGGACTCCT-3′ 5′-CCGACACGATGCACACTAAACATTACCCAA-3′ 5′-TTGGCATGGGGGCGACCTTCACCCTCGCCC-3′ 5′-CCAAAGTCAACCAACGGTACGACAGGGTCA-3′	[45]
*B. bigemina*	qPCR	18S rDNA	RTBbF/RTBbR	ND	5′- AGCTTGCTTTCACAACTCGCC -3′ 5′- TTGGTGCTTTGACCGACGACAT -3′	[51]
*B. bigemina*	qPCR	18S rDNA	Forward/reverse	ND	5′- AATAACAATACAGGGCTTTCGTCT -3′ 5′- AACGCGAGGCTGAAATACAACT -3′	[52]
*B. bovis*, and *B. bigemina*	PCR-RFLP	18S rDNA	PiroA/Piro B RE: *Msp*I *Box*I	400 bp 250, 150 bp 290, 110 bp	5′-AATACCCAATCCTGACACAGGG-3′ 5′-TTAAATACGAATGCCCCCAAC-3′	[47]
*B. bigemina* and *B. bovis*	qPCR	mitochondrialcytochrome b	Cbisg 1 and 2 Cbosg 1 and 2	88 bp 88 bp	5′-TGTTCCAGGAGATGTTGATTC-3′ 5′-AGCATGGAAATAACGAAGTGC-3 5′-TGTTCCTGGAAGCGTTGATTC-3′ 5′-AGCGTGAAAATAACGCATTGC-3′	[49,50]
*B. divergens*	PCR	18S rRNA	BAB GF2/GR2	559 bp	5′-GYYTTGTAATTGGAATGATGG-3′ 5′-CCAAAGACTTTGATTTCTCTC-3′	[55]
*Bovine Babesia* spp.	PCR	18S rRNA	ND	422–440 bp	5′-GTTTCTGMCCCATCAGCTTGAC-3′ 5′-CAAGACAAAAGTCTGCTTGAAAC-3′	[56]
*B. divergens*	PCR	18S rRNA	ND	353 bp	5′-GTTTCTGMCCCATCAGCTTGAC-3′ 5′-CAATATTAACACCACGCAAAAATTC-3′	[56]
*Babesia* sp. *genotype EU1*	PCR	18S rRNA	ND	362 bp	5′-GTTTCTGMCCCATCAGCTTGAC-3′ 5′-AGACAAGAGTCAATAACTCGATAAC-3′	[56]
*B. orientalis*	Semi-nested PCR	18S rRNA	P1/B-R2 B-P2/B-R2	ND 257 bp	5′-AACCTGGTTGATCCTGCCAGTAGT-3′ 5′-CACACGCACAACGCTGAA-3′ 5′-TGAGAAACGGCTACCACA-3′ 5′-CACACGCACAACGCTGAA-3′	[57]
*B. occultans*	PCR/RLB hybridization	18S rRNA (V4 variable region)	RLB-F2/RLB-R2 Probe	460 bp	5′-GACACAGGGAGGTAGTGACAAG-3′ 5′-biotin-CTAAGAATTTCACCTCTGACAGT-3′ 5′-GTGTGCCTCTTTTGGCCCATC-3′ Species-specific containing a C12 amino linker in 5′	[60]
*B. ovata*	PCR	*β-tubulin*	ND	ND	5′-ACACTGTGCATCCTCACCGTCATAT-3′ 5′-CTCGCGGATCTTGCTGATCAGCAGA-3′	[61]
*B. vogeli*	PCR-RFLP	18S rRNA	PiroA/PiroB RE: *TaqI*	400 bp 203, 171, 26 bp	5′-AATACCCAATCCTGACACAGGG-3′ 5′-TTAAATACGAATGCCCCCACC-3′	[46,70,83]
*B. vogeli*	Semi-nested PCR	18S rRNA	455-459/793-772 BCV/793-772	339 bp 192 bp	5′-GTCTTGTAATTGGAATGATGGTGAC-3′ 5′-ATGCCCCCAACCGTTCCTATTA-3 5′-GTTCGAGTTTGCCATTCGTT-3′ 5′- ATGCCCCCAACCGTTCCTATTA-3′	[68,70]
*B. vogeli*	PCR	18S rRNA	BCV-F/Ba721R	422 bp	5′- GTGTTCGAGTTTGCCATTCG-3′ 5′-CCCAGAACCCAAAGACTTTGATTTCTCTCAAG-3′	[79]
*B. vogeli*	PCR-RFLP	18S rRNA	BJ1/BN2 RE: *ApoI*	489 bp 367, 122 bp	5′-GTCTTGTAATTGGAATGATGG-3′ 5′-TAGTTTATGGTTAGGACTACG-3′	[71]
*B. vogeli and B. canis*	PCR	*cox*1	BFor/BvRev/BcRev	450 bp 750 bp	5′-GCATCTGGAATAGCTAGTGC-3′ 5′-CTGCTTCTAAACCAGAAGTG-3′ 5′-TGGAAATGACCTACAACATAC-3′	[75]
*B. gibsoni*	PCR	18S rRNA	PiroA/PiroB	408 bp	5′-AATACCCAATCCTGACACAGGG-3′ 5′-TTAAATACGAATGCCCCCACC-3′	[77]
*B. gibsoni* and *B. vogeli*	nPCR (non-specific)	18S ssrRNA	5-22F/1661R 455-479F/793-722R (generic primers)	293–338 bp	5′-GTTGATCCTGCCAGTAGT-3′ 5′-AACCTTGTTACGACTTCTC-3′ 5′-GTCTTGTAATTGGAATGATGGTGAC-3′ 5′-ATGCCCCCAACCGTTCCTATTA-3′	[78]
*B. gibsoni*, *B. vogeli, B. canis*	PCR/RLB hybridization	18S rRNA (V4 variable region)	RLB-F2/RLB-R2 Probes	460 bp	5′-GACACAGGGAGGTAGTGACAAG-3′ 5′-biotin-CTAAGAATTTCACCTCTGACAGT-3′ Oligonucleotides probes (species-specific) linked to N-terminal N-(trifluoracetamidohexyl-cyanoethyl, N, N-diisopropyl phosphoramidite [TFA])-C6 amino	[81,82,84,86]
*B. gibsoni* and *B. vogeli*	Multiplex PCR	18S rRNA	BAGI F/BAGI R BAB1 F/BAB4 R	590 bp 488 bp	5′- TTGGCGGCGTTTATTAGTTC-3′ 5′- AAAGGGGAAAACCCCAAAAG-3′ 5′- GTGAACCTTATCACTTAAAGG-3′ 5′- CAACTCCTCCAC GCAATCG-3′	[73,74]
*B. canis*	PCR	18S rRNA	BcW-A/BcW-B	500 bp	5′-CATCTAAGGAAGGCAGCAGG-3′ 5′- TTAATGGAAACGTCCTTGGC-3′	[87]
*B. canis*	PCR	18S rRNA	PiroA/PiroB	408 bp	5′-AATACCCAATCCTGACACAGGG-3′ 5′-TTAAATACGAATGCCCCCACC-3′	[69]
*B. canis*	PCR (non-specific)	18S rRNA	BJ1/BN2 (generic primers)	ND	5′-GTCTTGTAATTGGAATGATGG-3′ 5′-TAGTTTATGGTTAGGACTACG-3′	[72,88,89,90]
*B. canis*	PCR nPCR	18S rRNA	BS1/PiroC PiroA/PiroC	ND	5′-GACGGTAGGGTATTGGCCT-3′ 5′-CCAACAAAATAGAACCAAAGTCCTAC-3′ 5′-ATTACCCAATCCTGACACAGGG- 3′ 5′-CCAACAAAATAGAACCAAAGTCCTAC-3′	[92,93]
*B. venatorum*	PCR (non-specific)	18S rRNA	BJ1/BN2 (generic primers)	411–452 bp	5′-GTCTTGTAATTGGAATGATGG-3′ 5′-TAGTTTATGGTTAGGACTACG-3′	[72,98,99]
*B. odocoilei*	Semi-nested PCR	18S rDNA	Piro_18S_300F/Piro_18S_1688R Cocci_18S_595F/Piro_18S_1688R	1393 bp 1147 bp	5′-GACGGTAGGGTATTGGCCTA-3′ 5′-CGACTTCTCCTTCCTTTAAGTGATAAG-3′ 5′-CCGCGGTAATTCCAGCTCCAAT-3′ 5′-CGACTTCTCCTTCCTTTAAGTGATAAG-3′	[100]
*B. caballi*	nPCR	BC48	BC48F1/BC48R3 BC48F11/BC48R31	530 bp 430 bp	5′-ACGAATTCCCACAACAGCCGTGTT-3′ 5′-ACGATTTCGTAAAGCGTGGCCATG-3′ 5′-GGGCGACGTGACTAAGACATG-3′ 5′-GTTCTCAATGTCAGTGACATCCGC-3′	[105,106]
*B. caballi*	PCR/RLB hybridization	18S rRNA (hypervariable V4 region)	RLB-F2/RLB-R2 Genotypes A Genotypes B	ND	5′-GACACAGGGAGGTAGTGACAAG-3′ biotin-5′-CTAAGAATTTCACCTCTGACAGT-3′ 5′-GTTGCGTTGTTCTTGCTTTTTGCTT-3′ 5′-CGGGTTATTGACTTCGCTTTTTCTT-3′	[109]
*B. divergens/B. venatorum/B. microti*	PCR/RLB hybridization	18S rRNA	Bath-F/Bath-R (generic primers)	ND	5′-TAAGAATTTCACCTCTGACAGTTA-3′ 5′-ACACAGGGAGGTAGTGACAAG-3′	[113]
*B. motasi*	PCR (non-specific)	18S rRNA	BTH 1F/BTH 1R GF2F/GR2R (generic primers)	561 bp	5′-CCTGAGAAACGGCTACCACATCT-3′ 5′-TTGCGACCATACTCCCCCCA-3′ 5′-GTCTTGTAATTGGAATGATG-3′ 5′-CCAAAGACTTTGATTTCTCTC-3′	[114]
*B. motasi*	PCR (non-specific)	Cytochrome b (*COB*)	COB F/COB R (generic primers)	550 bp	5′-CCATAGCAATTAATCCAGCTA-3′ 5′-ACCTTGGTCATGGTATTCTGG-3′	[114]
*B. motasi*	PCR (non-specific)	Cytochrome c (*COX-3*)	COX3 F/COX3 R (generic primers)	552 bp	5′-TCAACAAAATGCCAATATGT-3′ 5′-AAGTGCATCTTTGGGAGAAG-3′	[114]
*B. microti*	nPCR	*β*-tubulin	Tubu93 F/Tubu897 R Tubu192 F/Tubu782 R	551 bp	5′-GAYAGYCCCTTRCAACTAGAAAGAGC-3′ 5′-CGRTCGAACGAACATTTGTTGHGTCARTTC-3′ 5′-ACHATGGATTCTGTTAGATCYGGC-3′ 5′-GGGAADGGDATRAGATTCACAGC-3′	[114]
*B. microti*	RT-PCR	18S rRNA	ND Probe	ND	5′-AACAGGCATTCGCCTTGAAT-3′ 5′-CCAACTGCTCCTATTAACCATTACTCT-3′ 6FAM-CTACAGCATGGAATAATGA-MGBNFQ	[115]
*B. duncani*	nPCR	*β-tubulin*	F34/R323 BtubFn/BtubRn	ND 175–181 bp	5′-TGTGGTAACCAGATYGGWGCCAA-3′ 5′-TCNGTRTARTGNCCYTTRGCCCA-3′ 5′-TCWGACGAGCACGGCATYGA-3′ 5′-CCAGGCTCCAARTCCATYAA-3′	[116]
*B. ovis*	PCR	ssrRNA	Bbo-F/Bbo-R	549 bp	5′-TGGGCAGGACCTTGGTTGTTCT-3′ 5′-CCGCGTAGCGCCGGCTAAATA-3′	[124,130]
*B. ovis*	qPCR (non-specific)	18S	Bab_18s_F/Bab_18s_R Bab_18s_P	ND	5′-TTGGGGGCATTCGTANTNRAC-3′ 5′-TTCTTGATTAATGAAAACGTCTTG-3′ 6FAM-AAGACGAACTACTGCGAAAGCATTTGC-TAMRA	[131]
*B. ovis*	PCR (non-specific)	18S rRNA	CRYPTOF/CRYPTOR	ND	5′-AACCTGGTTGATCCTGCCAGT-3′ 5′-GCTTGATCCTTCTGCAGGTTCACCTAC-3′	[131,132]
*B. ovis*	PCR qPCR	BoSPD BoSPD	SDP forward/SDP reverse SDP forward/SDP reverse	486 bp 141 bp	5′-ATGTTGGCCAAGTATCTTGCC-3′ 5′-CTACGTCAATTTGGCCTTGAACTC-3′ 5′-TAATGACGCAGACCTGATGG-3′ 5′-GTTTGATCACCCTCGGAAAC-3′	[125,133,134]

ND: data not defined; RE: Restriction enzyme.

## Data Availability

No new data were created or analyzed in this study. Data sharing is not applicable to this article.
